# Screening for poverty and related social determinants to improve knowledge of and links to resources (SPARK): development and cognitive testing of a tool for primary care

**DOI:** 10.1186/s12875-023-02173-8

**Published:** 2023-11-25

**Authors:** Itunuoluwa Adekoya, Alannah Delahunty-Pike, Dana Howse, Leanne Kosowan, Zita Seshie, Eunice Abaga, Jane Cooney, Marjeiry Robinson, Dorothy Senior, Alexander Zsager, Kris Aubrey-Bassler, Mandi Irwin, Lois Jackson, Alan Katz, Emily Marshall, Nazeem Muhajarine, Cory Neudorf, Andrew D. Pinto

**Affiliations:** 1 Upstream Lab, Li Ka Shing Knowledge Institute, MAP Centre for Urban Health Solutions, Unity Health Toronto, 30 Bond Street, Toronto, ON M5B 1W8 Canada; 2 Department of Family Medicine, Dalhousie University, Halifax, Canada; 3 Primary Healthcare Research Unit, Memorial University of Newfoundland and Labrador, St. John’s, Canada; 4 Department of Family Medicine, Rady Faculty of Health Sciences, University of Manitoba, Winnipeg, Canada; 5 Department of Community Health & Epidemiology, University of Saskatchewan, Saskatoon, Canada; 6 Faculty of Medicine, Memorial University, St. John’s, Canada; 7 School of Health and Human Performance, Dalhousie University, Halifax, Canada; 8 Manitoba Centre for Health Policy, University of Manitoba, Winnipeg, Canada; 9 Saskatchewan Population Health and Evaluation Research Unit, Saskatoon, Canada; 10 College of Medicine, University of Saskatchewan, Saskatoon, Canada; 11 Department of Family and Community Medicine, St. Michael’s Hospital, Toronto, Canada; 12 Department of Family and Community Medicine, Faculty of Medicine, University of Toronto, Toronto, Canada; 13 Dalla Lana School of Public Health, University of Toronto, Toronto, Canada

**Keywords:** Primary health care, Family medicine, Health equity, Healthcare disparities, Minority and vulnerable populations, Socioeconomic disparities in health, Social determinants of health, Validation study, Sociodemographics, Screening tool, Qualitative research

## Abstract

**Background:**

Healthcare organizations are increasingly exploring ways to address the social determinants of health. Accurate data on social determinants is essential to identify opportunities for action to improve health outcomes, to identify patterns of inequity, and to help evaluate the impact of interventions. The objective of this study was to refine a standardized tool for the collection of social determinants data through cognitive testing.

**Methods:**

An initial set of questions on social determinants for use in healthcare settings was developed by a collaboration of hospitals and a local public health organization in Toronto, Canada during 2011–2012. Subsequent research on how patients interpreted the questions, and how they performed in primary care and other settings led to revisions. We administered these questions and conducted in-depth cognitive interviews with all the participants, who were from Saskatchewan, Manitoba, Ontario, and Newfoundland and Labrador. Cognitive interviewing was used, with participants invited to verbalize thoughts and feelings as they read the questions. Interview notes were grouped thematically, and high frequency themes were addressed.

**Results:**

Three hundred and seventy-five individuals responded to the study advertisements and 195 ultimately participated in the study. Although all interviews were conducted in English, participants were diverse. For many, the value of this information being collected in typical healthcare settings was unclear, and hence, we included descriptors for each question. In general, the questions were understood, but participants highlighted a number of ways the questions could be changed to be even clearer and more inclusive. For example, more response options were added to the question of sexual orientation and the “making ends meet” question was completely reworded in light of challenges to understand the informal phrasing cited by English as a Second Language (ESL) users of the tool.

**Conclusion:**

In this work we have refined an initial set of 16 sociodemographic and social needs questions into a simple yet comprehensive 18-question tool. The changes were largely related to wording, rather than content. These questions require validation against accepted, standardized tools. Further work is required to enable community data governance, and to ensure implementation of the tool as well as the use of its data is successful in a range of organizations.

**Supplementary Information:**

The online version contains supplementary material available at 10.1186/s12875-023-02173-8.

## Background

The social determinants of health (SDoH) are the conditions in which we live, work, and play that impact individual and population health [1]. Our income, social status, education, as well as our physical environment are key social determinants of health [2]. While the relationship between social factors and health has been known for millennia [3], healthcare services have typically focused on biomedical solutions. Substantial evidence that improved access during the 20th century to healthcare services did not result in reduced health inequities [4, 5] but have led to research on root causes. Despite universal healthcare in Canada, income-related health inequities still persist in access to primary care services [6, 7]. In the World Health Organization Commission’s 2008 Final Report, governments were urged to explore how healthcare systems can consider and address social determinants [1, 8–12]. Subsequently, the British Medical Association [11], the Canadian Medical Association [10], the College of Family Physicians of Canada [13], the American College of Physicians [12] and other bodies have developed guidelines to support health providers and health organizations to take action on social determinants of health.

Accurate SDoH data that is linked to, or part of an individual’s health records is essential to identify opportunities for action to improve health outcomes and address health inequities. For example, care teams can help address patient needs through referrals to relevant community resources [14]. Accurate SDoH data is also critical for identifying patterns of inequity at an organization or community level, for developing interventions to reduce health inequities and for evaluating the impact of those interventions [15]. Furthermore, individual-level SDoH data has been shown to be strong predictors of health outcomes [16]. For example, income, housing, employment, and education have been shown to predict 30-day hospital readmissions [17]. While social data is often elicited during clinical visits, this information varies between providers and is scattered in different parts of the health record. In most countries, few health providers or organizations routinely collect data in an organized way on the social determinants of health, beyond sex and age [15, 16, 18, 19]. Currently, there is no universal sociodemographic tool used across health organizations in Canada [20]. Having a standardized tool, with questions that have been refined and validated through testing with users of the healthcare system, is critical for collecting accurate social determinants data. Using a standardized tool to collect social determinants of health data across health organizations would enable data to be used at an aggregate level to identify inequities across and within populations and facilitate public policy aimed at tackling health inequities.

The objective of this study was to refine, validate and standardize a tool for the collection of robust social determinants data, building on previous work, and to conduct cognitive testing with a large group of diverse individuals through in-depth interviews.

## Materials and methods

Research ethics approval was obtained from Unity Health Toronto (#20–241), the University of Manitoba Health Research Ethics Board (#HS24204), the Newfoundland and Labrador Health Research Ethics Board (# 2020.259) and the Saskatchewan Behavioural Research Ethics Board (#2373).

### Background to the SPARK tool

An initial set of questions (called the Health Equity Questionnaire) on social determinants for use in healthcare settings was developed by a collaboration of hospitals and a local public health organization in Toronto, Canada during 2011–2012 [18]. Questions on language, immigration, race, ethnicity, religion, disability, gender identity, sexual orientation, income, and housing were constructed based on a review of the literature, meetings, and consultations. These questions were pilot tested with approximately 1600 individuals across four organizations [15, 18]. Subsequent research on how patients interpreted the questions, and how they performed in primary care [21–24] and other settings [20], led to revisions and the inclusion of additional domains including employment, medication access and social isolation. Following preliminary pilot testing of these revised questions in five primary care clinics in Ontario with a small number of participants, the questions were further refined, resulting in the Screening for Poverty And Related determinants to improve Knowledge of and links to local resources (SPARK) Tool. The SPARK Tool includes 16 questions that cover demographics (language, immigration status, Indigenous identity, race, disability status, sex at birth and gender identity, sexual orientation) and social needs (education, income, medication access, housing status, social isolation, transportation, cost of utilities, and precarious employment; see Appendix 1). This initial tool had not been validated, except specific questions such as the income (‘making ends meet’) and precarious employment questions. This study is part of a larger study to refine, update and validate the SPARK Tool through cognitive interviewing and psychometric testing. This paper focuses on the process of updating and validating the 16-item version of the SPARK Tool through cognitive interviewing.

### Data collection

One hundred and ninety-five (195) participants were recruited from four provinces in Canada: Saskatchewan, Manitoba, Ontario, and Newfoundland and Labrador. Data collection was conducted from April 2021 to January 2022. Study advertisements were circulated using social media, through classified advertising websites, using collaborator distribution lists, and with posters placed in waiting rooms in health centers and community spaces. Study advertisements were in English, French and the top three non-Indigenous languages spoken in participating provinces according to the latest national census [25, 26]: Arabic, Farsi, Punjabi, simplified Chinese, and Tagalog. The only inclusion criterion was that participants were aged 18 years or older. All interviews were conducted virtually, and informed consent was obtained from participants once they had read and understood the detailed information sheet provided. Interviews were multi-part and lasted approximately 1.5 h. Data on Indigenous identity and race were not collected in Manitoba, based on feedback from the local research ethics board on the potential negative impact on relationships with Indigenous communities.

Cognitive interviews were conducted by five members of the study team (ADP, DH, IA, LK, ZS) and two research assistants, who all identified as female. All interviewers had moderate to extensive experience with conducting qualitative interviews, but most were new to cognitive interviewing. Cognitive interviewing is a technique used to gain insight into learners’ perceptions in which individuals are invited to verbalize thoughts and feelings as they examine information [27]. In this study, surveys were self-administered in the presence of the interviewer. Participants were asked to read the survey question and options provided aloud and *think aloud*, by sharing their immediate thoughts on each question. Participants then selected their answer choice. Minimal unscripted concurrent probing was done by interviewers, except to prompt participants to continue to think aloud throughout the survey. Interviewers recorded the thoughts of participants for each question. Interviewers were trained to look out for: issues related to question wording, issues related to unclear question objective, questions with redundancy or repetitiveness, issues of burden or length of the survey, and questions that lacked response options participants that frequently want to select [28].

### Data analysis

**Fig. 1:**
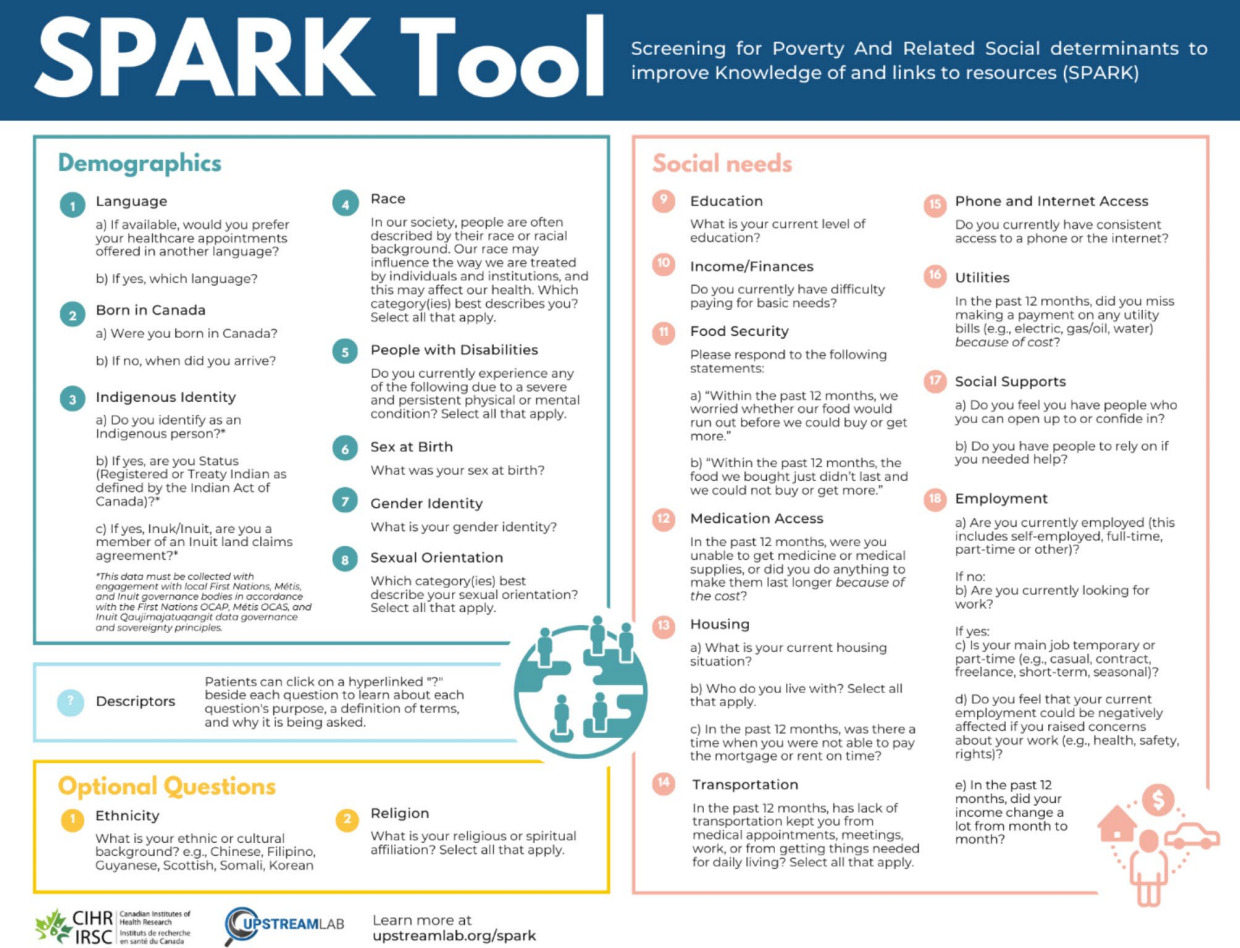
SPARK Tool for collecting data on social determinants in primary care

The analysis was conducted by the five members of the study team who had conducted the interviews. Informal analysis of the cognitive interviews was conducted as described by Willis [28].Using the interview notes, we thematically grouped unique topics or concerns that emerged for each question and assessed the frequency of each theme. Results were summarized by each question in a table with columns describing the topic or concern, and the frequency of each theme. Study team members reviewed the themes question-by-question and updated the SPARK Tool to address topics or concerns (see Appendix 1). The team first prioritized question changes according to the frequency of the concerns raised, but reviewed all topics to ensure important concerns, even if mentioned infrequently, were addressed. For example, a concern only mentioned once or twice would warrant a question change if the researchers agreed that it might be more frequently stated in a larger sample, per Willis’ guidance [28]. This review and editing process was done in a series of meetings where study team members discussed each question, including all topics or concerns, as well as potential solutions suggested by the participants to determine how to improve the SPARK Tool. Decisions made for each question were recorded. Topics or concerns were addressed by either modifying question wording; adding, editing or removing answer options; removing or replacing the question; or generating a “descriptor” to explain why a question was being asked or to define terms in the question or response choices that were commonly misunderstood. Once a first draft was created, the tool was presented to the larger study team, comprised of researchers and patient partners, to provide feedback on the changes. The tool was also presented to an advisory committee that included subject matter experts on sociodemographic data. Feedback was received from other subject matter experts that were not part of the study team or advisory committee through emails and brief one-on-one meetings. Once all the feedback was incorporated, the SPARK Tool was finalized (see Fig. 1).

## Results

Three hundred and seventy-five individuals responded to the study advertisements and 195 ultimately participated in cognitive interviews: 125 (64.1%) from Ontario, 25 (12.8%) from Saskatchewan, 24 (12.3%) from Manitoba, and 21 (10.8%) from Newfoundland and Labrador. Although the study was advertised in other languages, all interviews were conducted in English, as no non-English speaking participants contacted the study team. Participants were diverse: 148 (76%) identified as non-white, 71 (36%) indicated not being born in Canada, and 38 (19.5%) noted they had difficulty making ends meet at the end of the month. Although the majority of participants reported a college or university degree (129, 66%), 8 (4%) individuals indicated they did not complete high school and 9 (5%) individuals had high school or equivalent (see Table 1). In the following sections, we discuss the topics that emerged from the cognitive interviews and decisions made to improve the SPARK Tool. An “at-a-glance” version of the final SPARK Tool questions is shown in Fig. 1. The full version of the final SPARK Tool with response options and descriptors is presented in Appendix 2.

**Table 1:** Demographics of participants

Characteristics*	N (%)
Language preference (translation)**	
Non-English/Non-French preference (translation would be helpful)	14 (7)
English/French preference (translation not needed)	178 (91)
No response	≤ 5
***Born in Canada***	
Yes	120 (62)
No	71 (36)
No response	≤ 5
Indigenous Identity***	
Yes - First Nations	8 (4)
Yes - Métis	≤ 5
Yes - Inuk/Inuit	≤ 5
No	157 (81)
Data not collected (MB)	24 (12)
No response	≤ 5
Race***	
Arab, Middle Eastern or West Asian	11 (6)
Black	17 (9)
East Asian	18 (9)
Indigenous (First Nations, Metis, Inuk/Inuit)	8 (4)
Latino/Latina/Latinx	≤ 5
South Asian or Indo-Caribbean	31 (16)
Southeast Asian	11 (6)
White	47 (24)
Another Race Category	6 (3)
Mixed Race	16 (8)
Data not collected (MB)	24 (12)
No Response	≤ 5
***Sex at Birth***	
Female	133 (68)
Male	58 (30)
Intersex	≤ 5
No response	≤ 5
***Gender Identity***	
Woman	126 (65)
Man	58 (30)
Transgender	≤ 5
Gender fluid or Gender nonbinary	8 (4)
No response	≤ 5
***Sexual Orientation***	
Heterosexual (‘straight’, male/female relationships or two different binary genders)	165 (85)
Gay	≤ 5
Lesbian	≤ 5
Bisexual	9 (5)
Queer or pansexual	10 (5)
Another	≤ 5
No response	≤ 5
***Education***	
Some grade school	≤ 5
Completed grade school (grade 1–8)	≤ 5
Some high school	≤ 5
Completed high school (grade 9–12)	9 (5)
Trades Certificate/Diploma	14 (7)
Some college/university	39 (20)
College/university degree	94 (48)
Postgraduate degree	34 (17)
No formal schooling	≤ 5
No response	≤ 5
***Housing***	
Own home	72 (37)
Rent	73 (37)
Staying with friends or relatives because you have no alternative [couch surfing]	10 (5)
Shelter	≤ 5
On the street	≤ 5
Other	36 (18)
No response	≤ 5
***Making ends meet***	
Yes	43 (22)
No	151 (77)
No response	≤ 5

*These demographics were collected as part of participants’ answers to the SPARK Tool questions

** Based on responses to the SPARK Tool question: If it could be arranged, would translation into another language be helpful at your next appointment?

*** Note that one of the study sites, Manitoba, did not collect any race or indigenous identity data. MB had 24 participants. These are recorded under “Data not collected (MB)”

### General perspectives on the SPARK tool

Throughout the SPARK Tool, many participants indicated they were unclear why some questions were asked in the context of receiving healthcare. Based on this feedback, we added descriptions to each question, to explain the purpose of the question and its potential use in care at an individual and organisational level. Many participants also indicated being unfamiliar with terms used, and descriptions also provide definitions.

For many questions about social needs, participants expressed confusion concerning the timeframe for and frequency of the social need they were being asked to indicate. For example, with the question, *“Do you have difficulty making ends meet at the end of the month?”*, 11 (6%) participants indicated they could make ends meet some months (frequency) or could regularly make ends meet a year ago but not currently (timeframe). To address this feedback, we updated the questions to include a specific timeframe (e.g., ‘in the past 12 months’) and, in some cases, updated answer options to include frequency (e.g., ‘always’ or ‘sometimes’).

For questions that had ‘not applicable’ as an option, a large number of participants were confused about the difference between ‘no’ and ‘not applicable’. For example, 19 (10%) participants were unclear how ‘not applicable’ was defined for the employment question, and 14 (7%) participants that were unclear for the medication question. Therefore, we qualified the ‘not applicable’ option with a short phrase. For example, for the question about paying one’s rent or mortgage on time, we replaced ‘not applicable’ with ‘not applicable, I do not have to pay rent or mortgage’.

For certain questions, such as those concerning whether the participant was born in Canada, sex at birth, gender identity, and sexual orientation, several participants noted they may be unwelcome or uncomfortable to complete. To address this concern, the research team included a ‘prefer not to answer’ option to all the questions in the final survey.

### Additional and optional questions

Based on feedback from study participants, research staff, patient partners, advisory committee members, and other subject matter experts, additional domains were added to the questions on social needs, including questions on food security, and phone and internet access. Ethnicity and religion were added as optional questions. These optional questions should be used at the discretion of healthcare leaders if feel that they are necessary for use at their respective healthcare organizations.

### Perspectives on the separate SPARK tool questions

#### Language preference for healthcare and need for translation

The wording of the question concerning language needs, *“If it could be arranged, would translation into another language be helpful at your next appointment?”*, was unclear for only seven (3.6%) participants. Five (2.6%) participants were unsure if the question was referring to a healthcare appointment or any appointments. To clarify, we added ‘healthcare’ to the updated question. In addition, to reduce data entry error, we added a list of optional drop-down answer choices for language preferred.^1^

#### Born in Canada

Among 71 (36%) participants that were not born in Canada, three participants could not recall what year they arrived in Canada. Given that this question is aimed at determining newcomer status, we revised the answer options to include ‘less than 5 years’, ‘5 to 9 years’ and ‘10 years or more’ instead of an open-text field. This reduces data entry error and addresses recall issues. It also fits with Statistics Canada’s definition of a recent immigrant or newcomer.^2^ A limitation of this question is that it does not account for people who arrived more than once in Canada. Three (1.5%) participants expressed confusion, indicating that they moved to Canada more than once.

#### Indigenous identity

In this paper, we have not reported cognitive interview results from study participants on this question as we believe that the reporting of these results should be governed and led by Indigenous communities. We have included in the descriptor a statement that this data must be collected with engagement with local First Nations, Métis, and Inuit governance bodies in accordance with the First Nations OCAP, Métis OCAS, and Inuit Qaujimajatuqangit data governance and sovereignty principles.^3^

#### Race

The race question includes a preamble intended to explain why race is being asked about at a healthcare appointment. Twelve (6.1%) participants indicated that the preamble was too technical or overwhelming or requested simpler language. Eight (4.1%) participants noted that ‘not based in science’ is technically incorrect or unnecessary (e.g., although race is a social construct, many scientific disciplines have studied and linked differences in skin color (e.g., melanin) to the concept of race, and some noted that ‘not based in biology’ is a more appropriate phrase. In addition to suggested edits to the preamble, eighteen (9.2%) participants requested examples of countries or regions for each race category in the answer options, which we have now included. Fifteen participants wanted the ‘White’ and ‘Black’ option to be separated into different “types” of White or Black people. For example, participants suggested splitting the ‘Black’ option into African, Afro-Caribbean, African American etc. However, based on the definition of race and ethnicity, which we describe in the newly added descriptors, “types” of white or black people would correspond with ethnicity. The revised SPARK Tool provides an optional ethnicity question to address this topic. Eight (4.1%) participants indicated preference for an ethnicity question instead of a race question. The participant feedback on this question was valuable, informed the creation of, and changes to other questions. However, we did not edit this particular question as changes to the race question itself were not strongly indicated in light of the cognitive interviews and the existing question aligned closely with existing national standards for race-based data [33].

#### Disability status

For 38 (19.5%) participants, the disability question, *“In general, do you experience any of the following due to a physical, mental, or emotional condition?”*, and its answer options were seen as too broad and subject to interpretation. For example, ‘difficulty communicating’ may refer to a speech impairment or language barriers. Some participants (34, 17.4%) were unsure if a difficulty they experienced should be included. More specifically, there were 12 participants (6.1%) who were unsure if they should say they have a difficulty if it could be corrected with assistance (e.g., glasses for ‘difficulty seeing’). Based on the feedback we received, the study team revised the question to include ‘due to a severe and persistent physical, mental or emotional condition’. Additionally, the study team revised the answer options based on participant feedback. For example, it was unclear what ‘self-care’ meant to 19 (9.7%) participants, so we changed that option to ‘personal hygiene’. Including examples in the answer options and adding a descriptor helped to clarify that this question is intended to identify conditions that are severe and disabling. Eleven (5.6%) participants felt the options provided were limited and did not account for difficulties associated with having a mental health condition. The addition of answer options to include ‘difficulty with activities for daily living’ captures difficulties mentioned in the cognitive interviews that were not present in the original answer option.

#### Sex at birth

For the question, *“What was your sex assigned at birth?”*, six (3.1%) participants noted that ‘intersex’ status is not necessarily always ‘assigned’ at birth, therefore ‘assigned’ was removed from the question.

#### Gender identity

For the question, *“What is your current gender identity?”*, based on feedback from subject matter experts, the term ‘current’ was removed from the question as it was deemed redundant or unnecessary for answering the question. Nine (4.6%) participants felt the options provided for gender identity were limited. Based on feedback from the participants as well as from subject matter experts, additional options, including ‘transgender woman’ and ‘transgender man’ were included in the question. For five (2.6%) participants, the difference between ‘sex at birth’ and ‘gender identity’ was unclear, and hence, the descriptor includes an explanation.

#### Sexual orientation

Two additional options, ‘demisexual’ and ‘asexual’, were added to the sexual orientation question (‘which best describes your sexual orientation?’) as 17 (8.7%) participants felt that the original options were too limited, and a few participants specifically mentioned those options should be included. This change was supported by the study team’s review of the literature and consultations with subject matter experts. We removed the definition in the ‘heterosexual’ option (‘male/female relationships or two different binary genders’) based on feedback from 16 (8.2%) participants who felt it was unnecessary, some of whom found it offensive. ‘Queer’ and ‘pansexual’ were separated into two different options as 9 (4.6%) participants highlighted that these terms are not synonymous. Some participants (2, 1.0%) indicated that they felt that gender could be represented by more than one answer option (e.g., queer and bisexual), therefore individuals can now select multiple options.

#### Education

For the education question, *“What is the highest level of education you have completed?”*, three (1.5%) participants and a patient partner on our study team felt that the phrase ‘*highest* level of education’ in the question was un-inclusive, hierarchical, elitist, or offensive, so the term ‘highest’ has been removed. Twenty-one (10.8%) participants mentioned that the education answer options were too limited and, in some cases, confusing. Therefore, several options were added or revised including replacing ‘trades certificate/diploma’ and ‘college/university degree’ with ‘college, CEGEP or other non-university certificate or diploma’, ‘completed registered apprenticeship or other trades certificate or diploma’ and ‘undergraduate degree’. Examples were added to clarify the term ‘postgraduate’ as 14 (7.2%) participants, many of which spoke English as a Second Language (ESL) or completed their education outside of Canada, were unfamiliar with the term. We clarified or removed the word ‘some’ in the options and replaced with ‘ongoing’ in most cases. One limitation to the updated question is that participants are not able to select more than one option for their level of education as requested by 9 (4.6%) participants. For example, in instances where a participant has attended both college and university and considers both education levels to be equivalent, they are forced to pick just one. Multiple choice selection was not added to this question as its purpose is to ascertain literacy level indicated by education level.

#### Finances

We changed the question from *“Do you have difficulty making ends meet at the end of the month?”* to *“Do you currently have difficulty paying for basic needs?”* as 26 (13.3%) participants, many of whom were ESL speakers, found the term ‘making ends meet’ difficult to understand. For 7 (3.6%) participants, it was unclear how to define a basic or essential need, so we included clarification in the descriptor. One limitation to the updated question is that it does not capture people who are able to pay for basic needs like food, shelter, clothing, but still have serious financial difficulties. This concern was identified by 7 (3.6%) participants. This brief question may not accurately capture every aspect of a person’s financial situation. However, it has been validated as a good predictor of poverty and identifying individuals living below the “poverty line” or low-income cut-off (LICO) [34].

#### Access to prescription medication insurance

Three (1.5%) participants felt that the question about medication access (‘in the last 12 months, did you avoid filling a prescription or do anything to make a prescription last longer because of the cost?’) did not consider medical devices or supplies (e.g., for diabetes management), as well as over-the-counter (OTC) medications, which may not be prescribed. To account for patients’ diverse medical needs, ‘prescription’ was replaced with ‘medication or medical supplies’.

#### Housing

We changed the question from *“What is your current housing?”* to *“What is your current housing situation?”* as the previous wording of the question was cumbersome according to 6 (3.1%) participants. There were no adequate options for 35 (17.9%) participants who lived with family or friends by choice (and not because of a lack of alternatives). To address this, we replaced the ‘own home’ and ‘rent’ options with ‘a place you or your family owns’ and ‘a place you or your family rents’. Some participants found the terms ‘couch surfing’ (12 participants) and ‘on the street’ (9 participants) stigmatizing, inappropriate or offensive. We replaced ‘couch surfing’ with ‘staying in someone else’s place because you have no alternative’ and ‘On the street’ with ‘Experiencing homelessness’. Nineteen (9.7%) participants felt that the options were limited and did not capture other living situations such as transitional housing or a nomadic lifestyle. We added additional options including supportive housing or group home, long-term care facility and correctional facility. We added “social housing, subsidized housing or rent-geared-to-income’ as an option to this question and therefore removed the follow-up question “Is your current housing social housing, subsidized housing or rent-geared-to-income?” from the SPARK Tool. Seven (3.6%) participants indicated wanting to be asked about their living arrangements, specifically about who they live with. For example, if they rent, to specify that they live with roommates. One participant mentioned that the ‘rent’ option is a wide category that could include living with multiple roommates or alone in a “big fancy apartment”. We added a follow-up question asking, “who do you live with? select all that apply”. For 18 (9.2%) participants, the options were inadequate and did not capture people who did not have to pay rent or people who were only able to make partial payment of the rent. ‘Not applicable, I do not have to pay rent or mortgage’ was added to address the first problem. The updated question does not yet capture participants who were only able to make partial rent payment, which is a limitation of the question.

#### Transportation

In the transportation question used in this study, *“In the past 12 months, did you avoid attending an important appointment because of the cost of transportation?”*, it was unclear to four (2.0%) participants what type of appointment the question was referring to. This has been clarified in the updated question. The updated question was adapted from the American Academy of Family Physicians (AAFP) Social Needs Screening Tool and the PRAPARE Toolkit [35, 36].

#### Utilities

Eleven (5.6%) people were unsure what constituted a utility bill, therefore, in the revised question and the created descriptor, we provided examples of utilities for clarity. The descriptor explains ‘the specific utilities you pay for may depend on where you live’, indicating that electric, gas/oil and water bills are only examples of utility bills. There were no adequate options for 8 (4.1%) participants whose utilities are included in the rent. If these participants experienced difficulty paying a bill it would be captured in part three of housing question which asks the participant, “in the past 12 months, was there a time when you were not able to pay the mortgage or rent on time?” Further, we have expanded the ‘not applicable’ answer option to state “not applicable, I did not have to pay utility bills in the past 12 months or utilities already included in rent”.

#### Social supports

Social support is captured by two questions, both of which were moved to follow the utilities question (initially placed after housing) as some participants found the transition from housing to social supports abrupt. For the first question, ‘do you feel you have family or close friends who you can open up to?’, 10 (5.1%) participants, many of whom were ESL speakers, the term ‘open up to’ was difficult to understand. We therefore revised the question adding ‘confide in’ for clarity (e.g., ‘do you feel you have people who you can open up to or confide in’). Six (3.1%) participants felt unable to provide a *yes* or *no* response to social support questions, explaining that they may not be able to open up all the time or may be able to open up about some things but not others. To address this, we added ‘yes, always, or sometimes’ to the ‘yes’ option. In addition, ‘family or close friends’ was replaced with ‘people’ in the question as three participants said they may be able to open up to people not in those categories, e.g., pastor, teacher, etc. For the follow-up question, ‘are you able to rely on them if you need help (e.g., transportation, emotional or financial assistance)?’, 32 (16.4%) participants, felt the categories or examples provided in the question were too specific. For example, participants may be able to rely on the people they referred to in the first question emotionally but not financially. We removed the examples and left the question open to any kind of help. Some of these categories, e.g., transportation, are addressed in other questions.

#### Employment

The SPARK Tool administered in this study included three questions aimed at identifying individuals experiencing precarious employment. However, 34 (17.4%) participants felt unable to answer the employment questions which were not inclusive of all employment situations, including full-time, permanent, self-employed, did not capture people in between jobs or working multiple jobs, and failed to capture participants that were retired. To address this, we added two questions: ‘are you currently employed?’ and ‘are you currently looking for work?’ before asking the follow up questions about precarious employment. These revisions will capture individuals unemployed and looking for employment and therefore may help connect people to employment resources. Among the precarious employment questions, the first of which asked, ‘are you employed in a casual, short-term, or temporary position?’, 12 (6.1%) participants were not familiar with the terms ‘casual’ and ‘short-term’. The umbrella terms for precarious work ‘temporary’ or ‘part-time’ were used instead. Examples of types of temporary or part-time employment were also included. Based on feedback from 4 (2.0%) participants who had multiple jobs, this question was revised to include ‘main job’ in the question to clarify that this is referring to primary source of employment. For the second precarious employment question, ‘do you feel fearful that you could be fired if you raise employment concerns?’, the phrase ‘raise employment concerns’ was confusing for 4 (2.0%) participants. To clarify this, we added examples of types of employment concerns. The question has also been modified to simplify the language. Based on feedback from research staff, ‘fired’ was replaced with ‘negatively affected’ in the question to account for people who may not be fired but endure negative consequences if or when they raise concerns. For the question about income instability, ‘does your pay vary a lot from month to month?’, we replaced ‘vary’ with ‘change’ for 12 (6.1%) participants who wanted the question reworded. Several of these participants also mentioned that their pay may change a lot due to other factors unrelated to precarious employment such as self-employment or shift work (e.g., nurses). This is a limitation that the updates have not addressed.

## Discussion

In this study, we conducted cognitive interviews with 195 diverse individuals from across four Canadian provinces to refine and develop a standard tool for collecting data on social determinants within primary care settings. For many participants, the value of this information being collected in typical healthcare settings was unclear, and hence, we included descriptors for each question. In general, the questions were understood, but participants highlighted a number of ways the questions could be changed to be even clearer and more inclusive. The findings from this study led to numerous ways to refine the questions.

Our findings are similar to other research on collecting sociodemographic data and screening for social needs in healthcare settings. Several previous studies have found that it is essential to clarify the reason for collecting data on social determinants at the outset [37–39]. Our findings regarding participants’ interpretation of the question on race fits our past research on an earlier version of this question [23], and confirms that the majority of individuals can identify an option that fits their self-identified race. Regarding the question on disabilities, our revised question is an improvement on an older version that contained a mixture of diagnoses and confusing terms [24], and fits with work by Morris et al., on a patient-centred disability status question [40]. Our findings on the questions related to gender identity and sexual orientation fit previous work [22], and suggest that new terms will continue to emerge that will need to be added as answer options to these questions.

The SPARK Tool fills a gap for a validated, national standardized SDoH tool for use in health care settings in Canada and beyond. The SPARK Tool is designed to be self-administered by patients, allowing patients to share their own social needs and therefore, improving the quality and validity of the SDoH data collected. Other standardized SDoH tools such as the PRAPARE Tool, which was developed for the United States context, is being implemented in health care settings across the United States and includes a wide range of SDoH domains [36]. One of the PRAPARE tool domains, transportation, was adapted in the SPARK Tool. Other tools are being developed such as the Accountable Health Communities Health-Related Social Needs screening tool [41], Health Leads Screening Toolkit [42], Upstream Risk Screening Tool [29, 39], have varying SDoH domains included, have been developed for specific national context or have different levels of validation. The extensive cognitive interviewing done with a large, diverse group of users of the Canadian health care system makes the SPARK Tool unique, as questions have been refined to be easily comprehended by all respondents including ESL speakers.

In addition to making the tool easier to understand and complete, two other goals were top of mind as we interpreted participants’ cognitive interviewing data and made adjustments to the tool. First, to make the questions inclusive and inviting so that all respondents see themselves and their needs and circumstances reflected, and second, to offer sufficient information in the questions and descriptors to convey the relevance and importance of collecting SDoH data in healthcare settings. By accomplishing all three objectives in our revisions to the tool, we believe patients will better understand its purpose, be more receptive to it, and more inclined to complete it. Patient perspectives on the collection of SDoH data, including their thoughts on how it is explained and the questions that are asked, were also collected during in-depth interviews that followed the cognitive interviews. Those results will be presented in forthcoming manuscripts. Moreover, the SPARK Tool is currently undergoing further validation through psychometric testing, including concurrent validity through comparison against other standardized tools. Finally, an ongoing study in which the SPARK Tool is being implemented in five clinics, one in each of Saskatchewan, Manitoba, Ontario, Nova Scotia and Newfoundland and Labrador, is examining the feasibility and acceptability of administering the tool in active primary care settings and will offer additional insight into potential for this tool in identifying and addressing SDoH in healthcare. This study is nearing completion and results will be published soon.

A limitation of our study is that all interviews were conducted in English, despite advertising in numerous languages and offering translation services if required. Future research is needed to determine whether translations of these questions perform well or elicit new or different concerns from patients. The exclusion of cognitive interview results for the Indigenous identity question in this study is a limitation and a gap remains in our understanding of Indigenous perspectives on collecting SDOH data in healthcare settings. As noted above, because the research team did not have established relationships with relevant Indigenous leaders and groups in the various participating provinces, and had not adequately engaged them in the design of this study, we decided to omit these results in keeping with the guidance put forward in First Nations OCAP, Métis OCAS, and Inuit Qaujimajatuqangit data governance and sovereignty principles [29–32]. To reduce the risks and potential harms of inappropriate data use and disclosure, Indigenous communities must be engaged as early as possible in the design and conduct of research and/or health data collection to guide and oversee the respectful and appropriate collection, storage, ownership, and use of their information. Several resources to guide researchers and practitioners in following these principles were included in the final SPARK Tool, and we encourage those who plan to administer the SPARK Tool or similar questionnaire to follow them closely. Finally, a potential limitation of this study is that during the virtual interviews, because participants were not *required* to have their cameras turned on, a few opted to not appear on video and non-verbal cues during their cognitive interviews may have been missed by the researcher.

## Conclusions

In this work we have refined a set of 16 sociodemographic and social needs questions into a simple yet comprehensive and inclusive, 18-question tool that can be used in healthcare organizations to collect data on sociodemographic and social needs. The changes that were required were largely relating to wording, rather than content and demonstrated that consensus can be achieved. These questions require further validation against accepted, standardized tools, and a future validation study is planned. Further work is required to enable community data governance [43], and to ensure implementation of the tool and well as use of its data is successful in a range of organizations.

### Electronic supplementary material

Below is the link to the electronic supplementary material.

Supplementary Material 1

Supplementary Material 2

## Data Availability

The underlying dataset is available from the authors upon reasonable request.

## Footnotes

1 The options are based on the top 20 languages and top five Indigenous languages spoken most often at home in Canada based on the 2016 Statistics Canada Census data [25, 26].

2 Statistics Canada’s definition of a recent immigrant or newcomer refers to a person who obtained a landed immigrant or permanent resident status up to five years prior to a given census year [28].

3 The descriptor includes references for First Nations OCAP, Métis OCAS, and Inuit Qaujimajatuqangit data governance and sovereignty principles [29–32].
