# Supplementary material for: Screening for poverty and related social determinants to improve knowledge of and links to resources (SPARK): development and cognitive testing of a tool for primary care
Source: BMC Prim Care. 2023 Nov 25;24:247. doi: 10.1186/s12875-023-02173-8 (PMC10675961; doi:10.1186/s12875-023-02173-8)
Supplement: Supplementary file 1 — Supplementary Material 1 [file 12875_2023_2173_MOESM1_ESM.docx]

**Appendix 1: Original and Updated SPARK Tool Questions**

| **Domain** | **Question Used in this Study** | **Updated Question** |
| --- | --- | --- |
| **Language preference** | **If it could be arranged, would translation into another language be helpful at your next appointment?**   - Yes - No   If yes, which language ________________ | **a) If available, would you prefer your healthcare appointments offered in another language?**   - - Yes   - No   - Do not know   - Prefer not to answer   b) **If yes, which language?** (drop-down menu)   - Options include top 20 non-official languages and top 5 Indigenous languages most spoken most often at home in Canada based on the 2016 Statistics Canada Census data |
| **Born in Canada** | **Were you born in Canada?**   - Yes - No   If no, what year did you arrive in Canada? ___________ | **a) Were you born in Canada?**   - - Yes   - No   - Do not know   - Prefer not to answer   **b)** **If no, when did you arrive?**   - less than 5 years ago - 5 to 9 years ago - 10 years ago or more |
| **Indigenous identity** | **Do you identify as First Nations, Métis and/or Inuk/Inuit**?   - Yes, First Nations - Yes, Métis - Yes, Inuk/Inuit - No | **a) Do you identify as an Indigenous person? Select all that apply:**   - Yes, First Nations - Yes, Métis - Yes, Inuk/Inuit - Yes, another Indigenous identity (specify) ____________ - No - Do not know - Prefer not to answer   b) **If yes, are you Status (****Registered or Treaty Indian as defined by the *Indian Act* of Canada)?**   - Yes, Status Indian (Registered or Treaty) - No - Not applicable, I am not an Indigenous person [remove if skip logic used] - Do not know - Prefer not to answer   c) **If yes, Inuk/Inuit, are you a member of an Inuit land claims agreement?**   - Yes - No - Not applicable, I am not an Indigenous person [remove if skip logic used] - Do not know - Prefer not to answer |
| **Race** | Preamble: **In our society, people are often described by their race or racial background. These are not based in science, but our race may influence the way we are treated by individuals and institutions, and this may affect our health.**  **Which category(ies) best describes you? Check all that apply:**   - Arab, Middle Eastern or West Asian - Black - East Asian - Indigenous (First Nations, Metis, Inuk/Inuit)   - Do you identify as Two-Spirit? Yes/No - Latino/Latina/Latinx - South Asian or Indo-Caribbean - Southeast Asian - White - Another race category: ________________ | **In our society, people are often described by their race or racial background. Our race may influence the way we are treated by individuals and institutions, and this may affect our health. Which category(ies) best describes you? Select all that apply**:   - Black (e.g., African, African Canadian, Afro-Caribbean descent) - East Asian (e.g., Chinese, Japanese, Korean, Taiwanese descent) - Indigenous (e.g., First Nations, Métis, Inuk/Inuit) - Latin American (Hispanic or Latin American descent) - Middle Eastern (e.g., Arab, Persian, West Asian descent (e.g., Afghan, Egyptian, Iranian, Kurdish, Lebanese, Turkish)) - South Asian (e.g., South Asian descent (e.g., Bangladeshi, Indian, Indo-Caribbean, Pakistani, Sri Lankan) - Southeast Asian (e.g., Cambodian, Filipino, Indonesian, Thai, Vietnamese, or other Southeast Asian descent) - White (e.g., European descent) - Another race category (please specify) ________________ - Do not know - Prefer not to answer |
| **Disability status** | **In general, do you experience any of the following due to a physical, mental, or emotional condition? *(Select all that apply)***   - Difficulty seeing - Difficulty hearing - Difficulty walking or climbing - Difficulty remembering or with concentration - Difficulty with self-care - Difficulty with communicating - None of the above | **Do you currently experience any of the following due to a severe and persistent physical or mental condition? Select all that apply:**   - Difficulty seeing (e.g., severe vision impairment) - Difficulty hearing (e.g., severe hearing loss) - Difficulty with walking or climbing (e.g., severe mobility issues) - Difficulty remembering or with concentration (e.g., severe memory loss or disorientation) - Difficulty with personal hygiene (e.g., physically unable or lack motivation to shower) - Difficulty with activities for daily living (e.g., physically unable or lack motivation to: e.g., eat, get out of bed, work) - Difficulty with communicating (e.g., severe speech impairment, trouble generating words) - Difficulty with comprehension (e.g., severe learning disability, trouble understanding words) - None of the above - Do not know - Prefer not to answer |
| **Sex at Birth** | **What was your sex assigned at birth? *(check one)***   - Female - Male - Intersex | **What was your sex at birth?**   - Female - Male - Intersex - Do not know   Prefer not to answer |
| **Gender Identity** | **What is your current gender identity? *(check one)***   - Woman - Man - Transgender - Gender fluid or Gender nonbinary - Two-Spirit (Indigenous) - Another (Specify)__________ | **What is your gender identity?**   - Woman - Man - Transgender man - Transgender woman - Gender fluid or non-binary - Two-Spirit (a term by and for Indigenous peoples) - Prefer to self-describe______________ - Do not know - Prefer not to answer |
| **Sexual Orientation** | **Which best describes your sexual orientation?**   - Heterosexual (“straight”, male/female relationships or two different binary genders) - Gay - Lesbian - Bisexual - Queer or Pansexual - Two-Spirit (Indigenous) - Another (Specify)________ | **Which category(ies) best describe your sexual orientation? Select all that apply:**   - Asexual or Aromantic - Bisexual - Demisexual - Homosexual or Gay - Heterosexual or Straight - Lesbian - Pansexual - Queer - Two-Spirit (a term by and for Indigenous peoples) - Prefer to self-describe_____________ - Do not know - Prefer not to answer |
| **Education** | **What is the highest level of education you have completed?**   - Some grade school - Completed grade school (grade 1-8) - Some high school - Completed high school (grade 9-12) - Trades Certificate/Diploma - Some college/university - College/university degree - Postgraduate degree - No formal schooling | **What is your current level of education?**   - No formal schooling - Grade school (grade 1-8) - Some high school, but did not graduate - High school or high school equivalency certificate (grade 9-12) - Completed Registered Apprenticeship or other trades certificate or diploma (or ongoing) - College, CEGEP or other non-university certificate or diploma (or ongoing) - Undergraduate degree or some university - Postgraduate degree or professional designation (e.g., Master’s, PhD, MD) - Do not know - Prefer not to answer |
| **Finances** | **Do you have difficulty making ends meet at the end of the month?**   - Yes - No | **Do you currently have difficulty paying for basic needs?**   - Yes - No - Not applicable, I do not have to pay for basic needs - Do not know - Prefer not to answer |
| **Medication Access** | **In the last 12 months, did you avoid filling a prescription or do anything to make a prescription last longer *because of the cost*?**   - Yes - No - Not Applicable | **In the past 12 months, were you unable to get medicine or medical supplies, or did you do anything to make them last longer *because of the cost*?**   - Yes - No - Not applicable, I did not have to get any medicine or medical supplies in the past 12 months - Do not know - Prefer not to answer |
| **Housing** | **a) What is your current housing?**   - Own home - Rent - Staying with friends or relatives because you have no alternative [couch surfing] - Shelter - On the street - Other (Specify)_______________ | **a) What is your current housing situation?**   - A place you or your family owns - A place you or your family rents - Social housing, Subsidized housing, or Rent-geared-to-income - Supportive housing or Group Home - Long-term care facility - Correctional facility - Staying in someone else’s place because you have no alternative - Experiencing homelessness (e.g., shelter, living in a public place or vehicle) - Other (Specify)_______________ - Do not know - Prefer not to answer |
|  | **b) If you rent 🡪 Is your current housing social housing, subsidized housing, or rent-geared-to-income?**   - Yes - No - Not Applicable | **b)** **Who do you live with? Select all that apply:**   - Parent(s) or Guardian(s) - Spouse or Partner - Child(ren) - Grandparent(s) - Sibling(s) - Other family - Friends or Roommates - Paid caregiver or attendant - Alone - Other (Specify) ____________________ - Do not know - Prefer not to answer |
|  | **c) If own home/rent 🡪 During the last 12 months, was there a time when you were not able to pay the mortgage or rent on time?**   - Yes - No - Not Applicable | **c) In the past 12 months, was there a time when you were not able to pay the mortgage or rent on time?**   - Yes - No - Not applicable, I do not have to pay rent or mortgage - Do not know - Prefer not to answer |
| **Transportation** | **In the past 12 months, did you avoid attending an important appointment because of the cost of transportation?**   - Yes - No - Not Applicable | **In the past 12 months, has lack of transportation kept you from medical appointments, meetings, work, or from getting things needed for daily living? Select all that apply:**   - Yes, it has kept me from medical appointments or getting medicines - Yes, it has kept me from non-medical meetings, appointments, work, or getting things that I need - No - Not applicable, I did not need transportation for these activities in the past 12 months - Do not know - Prefer not to answer |
| **Utilities** | **In the past 12 months, did you miss making a payment on your electric, gas or other utilities bills *because of cost*?**   - Yes - No | **In the past 12 months, did you miss making a payment on any utility bills (e.g., electric, gas/oil, water) *because of cost*?**   - Yes - No - Not applicable, I did not have to pay utility bills in the past 12 months or utilities already included in rent - Do not know - Prefer not to answer |
| **Social Supports** | **a) Do you feel you have family or close friends who you can open up to?**   - Yes - No | **a) Do you feel you have people who you can open up to or confide in?**   - Yes, I always or sometimes have someone - No, I don’t have anyone - Do not know - Prefer not to answer |
|  | **b) Are you able to rely on them if you need help (e.g., transportation, emotional or financial assistance)?**   - Yes - No - Not Applicable | **b) Do you have people to rely on if you needed help?**   - Yes, I always or have someone - No, I don’t have anyone - Do not know - Prefer not to answer |
| **Employment** | **a)** **Are you employed in a casual, short-term, or temporary position?**   - Yes - No - Not Applicable | **a) Are you currently employed (this includes self-employed, full-time, part-time or other)?**   - Yes - No - Do not know - Prefer not to answer   **b)** **Are you currently looking for work?**   - Yes - No - Not applicable, I am currently employed [remove if skip logic is used] - Do not know - Prefer not to answer   c) **Is your main job temporary or part-time (e.g., casual, contract, freelance, short-term, seasonal)?**   - Yes - No - Not applicable, I am not currently employed [remove if skip logic is used] - Do not know - Prefer not to answer |
|  | **b)** **Do you feel fearful that you could be fired if you raise employment concerns?**   - Yes - No - Not Applicable | **d)** **Do you feel that your current employment could be negatively affected if you raised concerns about your work (e.g., health, safety, rights)?**   - Yes - No - Not applicable, I am not currently employed [remove if skip logic is used] - Do not know - Prefer not to answer |
|  | **c) Does your pay vary a lot from month to month?**   - Yes - No - Not Applicable | e) **In the past 12 months, did your income change a lot from month to month?**   - Yes - No - Not applicable, I am not currently employed [remove if skip logic is used] - Do not know - Prefer not to answer |
| **Additional Questions** | | |
| **Food Security** | **N/A** | **Please respond to the following statements:**  **a) “Within the past 12 months, we worried whether our food would run out before we could buy or get more”**   - Often True - Sometimes true - Never true - Do not know - Prefer not Answer   b) **“Within the past 12 months, the food we bought just didn’t last and we could not buy or get more.”**   - Often True - Sometimes true - Never true - Do not know - Prefer not Answer |
| **Phone and Internet Access** | **N/A** | **Do you currently have consistent access to a phone or the internet?**   - Yes, phone only - Yes, internet only - Yes, both - No - Do not know - Prefer not to answer |
| **Optional Questions** | | |
| **Ethnicity** | **N/A** | **What is your ethnic or cultural background?** e.g., Chinese, Filipino, Guyanese, Scottish, Somali, Korean __________________ |
| **Religion** | **N/A** | **What is your religious or spiritual affiliation? Select all that apply:**   - Agnosticism - Animism or Shamanism - Atheism - Baha’i Faith - Buddhism - Christian Orthodox - Christian, *not included elsewhere on this list* - Confucianism - Hinduism - Islam - Jainism - Judaism - Native Spirituality - Pagan - Protestant - Rastafarianism - Roman Catholic - Sikhism - Spiritual - Unitarianism - Zoroastrianism - Other (Specify) _____________________ - Not Applicable, I do not have a religious or spiritual affiliation - Do not know - Prefer not to answer |
