# Supplementary material for: Screening for poverty and related social determinants to improve knowledge of and links to resources (SPARK): development and cognitive testing of a tool for primary care
Source: BMC Prim Care. 2023 Nov 25;24:247. doi: 10.1186/s12875-023-02173-8 (PMC10675961; doi:10.1186/s12875-023-02173-8)
Supplement: Supplementary file 2 — Supplementary Material 2 [file 12875_2023_2173_MOESM2_ESM.docx]

| Domain **Appendix 2: Updated SPARK Tool with Skip Logic and Descriptors** | Question (if branching or skip logic* is not used) *Skip logic is highly recommended | | If Branching or Skip Logic is used | Descriptor* 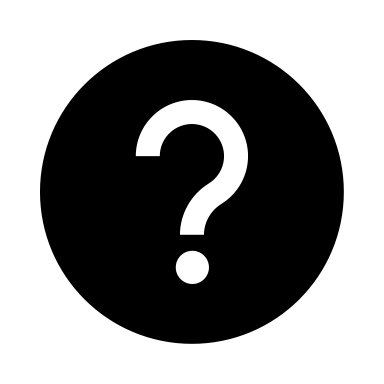*If possible, we recommend adding hyperlinked to the front of the question or to the front the option (for definitions), where applicable.  Include descriptor with *Why are we asking this question?* And *Understanding the question* with definition of sex and birth and gender identity  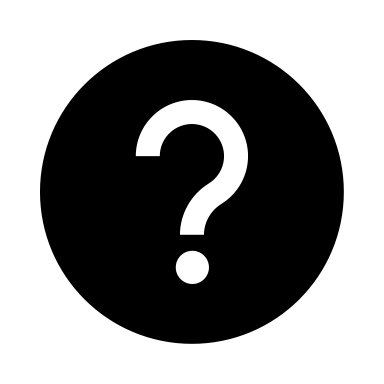For example:  What was your sex at birth?   - Female   Include definition of intersex   - Male - 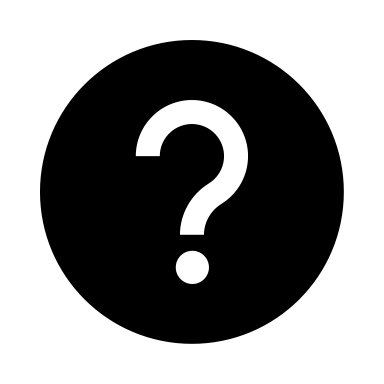Intersex - Do not know - Prefer not to answer |
| --- | --- | --- | --- | --- |
| Demographics | |  |  |  |
| 1: Language | **a) If available, would you prefer your healthcare appointments offered in another language?**   - - Yes   - No   - Do not know   - Prefer not to answer   b) **If yes, which language?** (drop-down menu)   - English [if applicable] - French [if applicable] - Arabic - Cantonese - Dene - German - Greek - Gujarati - Hindi - Inuktitut - Italian - Korean - Mandarin - Montagnais (Innu) - Ojib-Cree - Ojibway - Persian (Farsi) - Polish - Portuguese - Punjabi (Panjabi) - Romanian - Russian - Spanish - Tagalog (Pilipino, Filipino) - Tamil - Urdu - Vietnamese - Other (specify)___________ - Not applicable, I would not prefer healthcare appointments offered in another language [remove if skip logic used] | | **a) If available, would you prefer your healthcare appointments offered in another language?**   - - Yes → Go to question 1b   - No → Go to question 2   - Do not know → Go to question 2   - Prefer not to answer → Go to question 2   b) [If yes in 1a] **Which language?** (drop-down menu)   - English [if applicable] - French [if applicable] - Arabic - Cantonese - Dene - German - Greek - Gujarati - Hindi - Inuktitut - Italian - Korean - Mandarin - Montagnais (Innu) - Ojib-Cree - Ojibway - Persian (Farsi) - Polish - Portuguese - Punjabi (Panjabi) - Romanian - Russian - Spanish - Tagalog (Pilipino, Filipino) - Tamil - Urdu - Vietnamese - Other (specify)___________   Note: This represents the top non-Indigenous *languages most spoken at home* in Canada and top 5 Indigenous languages spoken in Canada [according to the 2016 Census](https://www12.statcan.gc.ca/census-recensement/2016/dp-pd/dt-td/Rp-eng.cfm?TABID=2&Lang=E&APATH=3&DETAIL=0&DIM=0&FL=A&FREE=0&GC=0&GID=1235625&GK=0&GRP=1&PID=110212&PRID=10&PTYPE=109445&S=0&SHOWALL=0&SUB=0&Temporal=2016&THEME=118&VID=0&VNAMEE=&VNAMEF=&D1=0&D2=0&D3=0&D4=0&D5=0&D6=0)  This list should be modified based on region. For example, using the top 20 *languages most spoken at home* in Ontario for SPARK Survey use in Ontario etc. | **Why are we asking this question?**  This information lets us know who may want an interpreter or translation services at our clinic.  This may also help us provide appropriate care to people who do not speak or read English or French. |
| 2: Born in Canada | **a) Were you born in Canada?**   - - Yes   - No   - Do not know   - Prefer not to answer   **b)** **If no, when did you arrive?**   - less than 5 years ago - 5 to 9 years ago - 10 years ago or more | | **a) Were you born in Canada?**   - - Yes → Go to question 2b below   - No → Go to question 3   - Do not know → Go to question 3   - Prefer not to answer → Go to question 3   b) [If yes in 2a] **When did you arrive?**   - less than 5 years ago - 5 to 9 years ago - 10 years ago or more | **Why are we asking this question?**  Providers may be able to refer people to appropriate newcomer services. This information lets us know who we serve at the clinic and may help us identify newcomers to Canada. |
| 3: Indigenous Identity | **a) Do you identify as an Indigenous person? Select all that apply:***   - Yes, First Nations - Yes, Métis - Yes, Inuk/Inuit - Yes, another Indigenous identity (specify) ____________ - No - Do not know - Prefer not to answer   *This data must be collected with engagement with local First Nations, Métis, and Inuit governance bodies in accordance with the First Nations OCAP, Métis OCAS, and Inuit Qaujimajatuqangit data governance and sovereignty principles | | **a) Do you identify as an Indigenous person? Select all that apply:***   - Yes, First Nations → Go to question 3b - Yes, Métis → Go to question 3b - Yes, Inuk/Inuit → Go to question **3b and 3c** - Yes, another Indigenous identity (specify) ____________ → Go to question 3b - No → Go to question 4 - Do not know → Go to question 4 - Prefer not to answer → Go to question 4   *This data must be collected with engagement with local First Nations, Métis, and Inuit governance bodies in accordance with the First Nations OCAP, Métis OCAS, and Inuit Qaujimajatuqangit data governance and sovereignty principles | **Why are we asking this question?**  Your provider may use this to refer you to First Nations, Métis or Inuit resources and supports. This information lets us know who we serve and helps us meet patient needs.    First Nations, Métis or Inuit identity data must be collected with local First Nations, Métis, and Inuit governance bodies. This way, the information is appropriately collected, stored, interpreted, used, or shared.^1-4^ This is in line with the First Nations OCAP, Métis OCAS, and Inuit Qaujimajatuqangit data governance and sovereignty principles.^1-4^  This information helps identify and address differences in health outcomes between First Nations, Métis and Inuit people and other racial groups in Canada.^5^  **Understanding the question:**  We ask “**Do you identify…**” in the question because we are asking you to tell us if you feel you are part of this group. This may include people who are Status or not Status.  **Truth and Reconciliation Commission of Canada** gives calls to action.^5^ These calls to action can improve outcomes for First Nations, Métis, and Inuit peoples in Canada.^5^  **Data sovereignty** means that each nation governs the collection, ownership, and application of its data. No matter where that data is stored^1-4^.  **Indigenous:** In Canada, Indigenous peoples are First Nations, Métis, and Inuit. These are the original people of the land that is now Canada.^6-9^  **First Nations** are the original people of the land that is now Canada. First Nations may or may not be Status (registered under the Indian Act) or live in a First Nations community.^10^  **Métis** have their own history, culture, language, and territory. Métis are people born in the Métis Nation Homeland (Manitoba, Saskatchewan, Alberta, as well as parts of Ontario, British Columbia, and the Northwest Territories).^11^  **Inuit** are the original people of the Northern region of Canada, Alaska, and Greenland. Their homeland included much of the land, water, and ice in the Artic.^7,12^   1. Canadian Institute for Health Information. (2020). [A path forward: toward respectful governance of First Nations, Inuit and Metis Data Housed at CIHI.](https://www.cihi.ca/sites/default/files/document/path-toward-respectful-governance-fnim-2020-report-en.pdf) Accessed April 14, 2022. 2. First Nations Information Governance Centre. [The First Nations principles of OCAP®](https://fnigc.ca/ocap). Accessed April 11, 2022. 3. University of Manitoba Faculty of Health Sciences. (2019) [Framework for Research Engagement with First Nation, Metis, and Inuit Peoples.](https://umanitoba.ca/faculties/health_sciences/medicine/media/UofM_Framework_Report_web.pdf) Accessed April 11, 2022. 4. Tagalik S. (2009–2010) National Collaborating Centre for Aboriginal Health[. Inuit Qaujimajatuqangit: The Role of Indigenous Knowledge in Supporting Wellness in Inuit Communities in Nunavut](https://www.ccnsa-nccah.ca/docs/health/FS-InuitQaujimajatuqangitWellnessNunavut-Tagalik-EN.pdf). Accessed April 11, 2022. 5. Truth and Reconciliation Commission of Canada. (2015) [Truth and Reconciliation Commission of Canada: Calls to Action.](http://nctr.ca/reports.php) Accessed April 11, 2022. 6. Statistics Canada. (2021)[. Indigenous group of person](https://www23.statcan.gc.ca/imdb/p3Var.pl?Function=DEC&Id=42924). Accessed April 11, 2022 7. The Canadian Encyclopedia. [Indigenous Peoples in Canada](https://www.thecanadianencyclopedia.ca/en/article/aboriginal-people) Accessed April 11, 2022 8. United Nations. [Indigenous Peoples at the United Nations](https://www.un.org/development/desa/indigenouspeoples/about-us.html) Accessed April 11, 2022 9. United Nations. [United Nations Declaration on the Rights of Indigenous Peoples](https://www.un.org/development/desa/indigenouspeoples/declaration-on-the-rights-of-indigenous-peoples.html). 2018 10. Government of Canada. (2014) [First Nations People in Canada.](https://rcaanc-cirnac.gc.ca/eng/1303134042666/1534961203322) Accessed April 11, 2022 11. Libraries and Archives Canada. Government of Canada. (2021) [First Nations People in Canada.](https://rcaanc-cirnac.gc.ca/eng/1303134042666/1534961203322) Accessed April 11, 2022 12. Government of Canada. (2021) [Inuit.](https://rcaanc-cirnac.gc.ca/eng/1100100014187/1534785248701) Accessed April 11, 2022 |
|  | b) **If yes, are you Status (Registered or Treaty Indian as defined by the *Indian Act* of Canada)?***   - Yes, Status Indian (Registered or Treaty) - No - Not applicable, I am not an Indigenous person [remove if skip logic used] - Do not know - Prefer not to answer   *This data must be collected with engagement with local First Nations, Métis, and Inuit governance bodies in accordance with the First Nations OCAP, Métis OCAS, and Inuit Qaujimajatuqangit data governance and sovereignty principles. | | b) [If yes, to any Indigenous option in 4a] **Are you Status (Registered or Treaty Indian as defined by the *Indian Act* of Canada)?**   - Yes, Status Indian (Registered or Treaty) - No - Do not know - Prefer not to answer   *This data must be collected with engagement with local First Nations, Métis, and Inuit governance bodies in accordance with the First Nations OCAP, Métis OCAS, and Inuit Qaujimajatuqangit data governance and sovereignty principles. | **Why are we asking this question?**  If you are Status, your provider may be able to refer you to more services.  You may be able to receive Non-Insured Health Benefits. This may include:   - some drugs - vision care - medical supplies - medical transportation   **Understanding the question:**  **Status (Registered or Treaty Indian)** is a person registered under the Indian Act of Canada. People with status may receive health services under the Non-Insured Health Benefits Program.^1,2^   1. Government of Canada. (2022). [About Indian Status](https://sac-isc.gc.ca/eng/1100100032463/1572459644986). Accessed April 14, 2022 2. Statistics Canada. [Registered or Treaty Indian status of person.](https://www23.statcan.gc.ca/imdb/p3Var.pl?Function=DEC&Id=42932) 2021. Accessed April 11, 2022 |
|  | c) **If yes, Inuk/Inuit, are you a member of an Inuit land claims agreement?***   - Yes - No - Not applicable, I am not an Indigenous person [remove if skip logic used] - Do not know - Prefer not to answer   *This data must be collected with engagement with local First Nations, Métis, and Inuit governance bodies in accordance with the First Nations OCAP, Métis OCAS, and Inuit Qaujimajatuqangit data governance and sovereignty principles. | | c) [If yes, Inuk/Inuit in 4a] **Are you a member of an Inuit land claims agreement?**   - Yes - No - Do not know - Prefer not to answer   *This data must be collected with engagement with local First Nations, Métis, and Inuit governance bodies in accordance with the First Nations OCAP, Métis OCAS, and Inuit Qaujimajatuqangit data governance and sovereignty principles. | **Why are we asking this question?**  If you are a member of an Inuit land claims agreement your provider may refer you to supports and services that may help you.  You may be able to receive Non-Insured Health Benefits. This may include:   - some drugs - vision care - medical supplies - medical transportation   **Understanding the Question:**  A member of an **Inuit land claims agreement** is a beneficiary of: Nunavut Land Claim Agreement, or Inuvialuit Final Agreement.  You may have been automatically registered and received your territorial health care card.  If you live outside of the land claim settlement area you can register with a land claim organization to receive benefits.   - - - 1. Government of Canada. [Your Health Benefits – A Guide for Inuit to Access Non-Insured Health Benefits](https://www.sac-isc.gc.ca/eng/1585310583552/1585310609830). Accessed April 22, 2022. |
| 4: Race | **In our society, people are often described by their race or racial background. Our race may influence the way we are treated by individuals and institutions, and this may affect our health. Which category(ies) best describes you? Select all that apply**:   - Black (e.g., African, African Canadian, Afro-Caribbean descent) - East Asian (e.g., Chinese, Japanese, Korean, Taiwanese descent) - Indigenous (e.g., First Nations, Métis, Inuk/Inuit)* - Latin American (e.g., Hispanic or Latin American descent) - Middle Eastern (e.g., Arab, Persian, West Asian descent (e.g., Afghan, Egyptian, Iranian, Kurdish, Lebanese, Turkish)) - South Asian (e.g., South Asian descent (e.g., Bangladeshi, Indian, Indo-Caribbean, Pakistani, Sri Lankan) - Southeast Asian (e.g., Cambodian, Filipino, Indonesian, Thai, Vietnamese, or other Southeast Asian descent) - White (e.g., European descent) - Another race category (please specify) ________________ - Do not know - Prefer not to answer   *This data must be collected with engagement with local First Nations, Métis, and Inuit governance bodies in accordance with the First Nations OCAP, Métis OCAS, and Inuit Qaujimajatuqangit data governance and sovereignty principles. | | Not Applicable | **Why are we asking this question?**  We are asking this question on race to help us know who we serve at the clinic. We’re interested in monitoring the experience our clients have in the clinic to ensure that everyone has a positive experience regardless of their racial background.  This information may also help us identify and address differences in health outcomes experienced by different racial groups.  **Understanding the question:**  **Race** is a term used to classify people into groups usually based on observable physical characteristics (for example, skin colour) but can also include characteristics such as accent or dress.^1,2,3^  Although these differences or groupings are created by the society we live in, racial categories can have significant consequences for people’s lives, including unfair and unjust treatment by individuals and institutions. This is what we call racism.  Race should not be confused with ethnicity. **Ethnicity** refers to groups of people who share a common culture or ancestry. They may also share a common language, religion, geographic origin, nationality, cultural traditions, migration history or other commonalites.^1,2^  **Mixed race** individuals are encouraged to select more than one racial group.  If you are unsure what to select, you can choose “Another” and specify what racial group(s) best describe you.  This question was adapted from the March 2022 CIHI Race-based standards.^1^   1. Canadian Institute for Health Information (2022) [Guidance on the Use of Standards for Race-Based and Indigenous Identity Data Collection and Health Reporting in Canada](https://www.cihi.ca/sites/default/files/document/guidance-and-standards-for-race-based-and-indigenous-identity-data-en.pdf) Ottawa, ON: CIHI. Accessed April 5, 2022 2. Torontohealthequity.ca. [Measuring Health Equity: Demographic Data Collection in Health Care](http://torontohealthequity.ca/wp-content/uploads/2018/03/Measuring-Health-Equity-Participant-Manual-2018.pdf). Accessed April 5, 2022 3. Government of Ontario Anti-Racism Directorate. [Data Standards for the Identification and Monitoring of Systemic Racism](https://www.ontario.ca/document/data-standards-identification-and-monitoring-systemic-racism). Accessed April 5, 2022 |
| 5: People with Disabilities | **Do you currently experience any of the following due to a severe and persistent physical or mental condition? Select all that apply:**   - Difficulty seeing (e.g., severe vision impairment) - Difficulty hearing (e.g., severe hearing loss) - Difficulty with walking or climbing (e.g., severe mobility issues) - Difficulty remembering or with concentration (e.g., severe memory loss or disorientation) - Difficulty with personal hygiene (e.g., physically unable or lack motivation to shower) - Difficulty with activities for daily living (e.g., physically unable or lack motivation to: e.g., eat, get out of bed, work) - Difficulty with communicating (e.g., severe speech impairment, trouble generating words) - Difficulty with comprehension (e.g., severe learning disability, trouble understanding words) - None of the above - Do not know - Prefer not to answer | | Not Applicable | **Why are we asking this question?**  Your provider may use this to inform the care you receive.  Your provider may also use this to refer you to appropriate services that may be helpful to you.  This information may help us provide better accommodations for people living with disabilities. And this can improve their overall access to health care.  **Understanding the question:**  **“Severe and persistent condition”** is about long-term conditions or disabilities that affect day-to-day activities.  The condition **does not have to be diagnosed** by your provider.  ***‘Difficulty seeing or hearing’*** is not asking about conditions that can be corrected with aids such as glasses or hearing aids.  ***‘Difficulty with communication’*** is asking about people who have this difficulty because of a physical or mental condition and not because of other things such as language barriers or reading or writing level. |
| 6: Sex at Birth | **What was your sex at birth?**   - Female - Male - Intersex - Do not know - Prefer not to answer | | Not Applicable | **Why are we asking this question?**  This information lets us know who we serve at the clinic and how well we are meeting patient needs. This information can also help us screen for and prevent some health conditions.  This question gives you a space to share your identity and to help avoid assumptions about you or your healthcare.    **Understanding the question:**  **Sex at Birth** is often recorded at a person’s birth and is usually based on a person’s reproductive system and other physical traits*.*^1^  While **Gender Identity** is a person’s sense of themselves relating to gender, be it male, female, a combination of both, or neither.^2^  Gender can be fluid and may change over time.^3^  **Intersex** refers to people born with differences in their reproductive features^2,4^ that may not be easily characterized as male or female.^5^   1. Statistics Canada, Government of Canada (2021).[Sex at birth of person](https://www23.statcan.gc.ca/imdb/p3Var.pl?Function=DEC&Id=24101) Accessed April 18, 2022 2. Human Rights Campaign (2011). [Glossary of Terms - Human Rights Campaign.](https://www.hrc.org/resources/glossary-of-terms)  Accessed April 18, 2022 3. Torontohealthequity.ca. [Measuring Health Equity: Demographic Data Collection in Health Care](http://torontohealthequity.ca/wp-content/uploads/2018/03/Measuring-Health-Equity-Participant-Manual-2018.pdf). Accessed April 18, 2022 4. Ucdavis.edu. (2014). [LGBTQIA Resource Center Glossary \| LGBTQIA Resource Center.](https://lgbtqia.ucdavis.edu/educated/glossary) Accessed April 18, 2022 5. Ohrc.on.ca. (2014). [Appendix B: Glossary for understanding gender identity and expression \| Ontario Human Rights Commission (ohrc.on.ca)](https://www.ohrc.on.ca/en/policy-preventing-discrimination-because-gender-identity-and-gender-expression/appendix-b-glossary-understanding-gender-identity-and-expression). Accessed April 18, 2022 |
| 7: Gender Identity | **What is your gender identity?**   - Woman - Man - Transgender man - Transgender woman - Gender fluid or non-binary - Two-Spirit (a term by and for Indigenous peoples) - Prefer to self-describe______________ - Do not know - Prefer not to answer | | Not Applicable | **Why are we asking this question?**  This information lets us know who we serve at the clinic and how well we’re meeting patient needs.  It helps us understand the range of gender identities that people at our clinic may experience.  This question gives you a space to share your identity and to help avoid assumptions about you or your healthcare.  **Understanding the question:**  **Gender Identity** is a person’s sense of themselves relating to gender, be it male, female, a combination of both, or neither.^1^  Gender can be fluid and may change over time.^2^  **Sex at Birth** is often recorded at a person’s birth and is usually based on a person’s reproductive system and other physical traits*.*^3^  **Gender non-binary** describes a person who may experience a gender identity that is neither exclusively woman or man, is a combination of woman and man or is between or beyond genders**.^4^**  Non-binary can also be used as an umbrella term for identities that are gender fluid, moving between and through identities.^1^  **Two-Spirit** is a term used by Indigenous peoples to self-describe and express diverse sexual, gender and/or spiritual identities.^5^ It is a way for Indigenous peoples to reconnect with their traditional languages, ways, and cultures.^6^  **Transgender** or trans is an umbrella term for people whose gender identity or expression is different from conventional or cultural expectations based on the sex they were assigned at birth.^7,8^   1. Ohrc.on.ca. (2014). [Appendix B: Glossary for understanding gender identity and expression \| Ontario Human Rights Commission.](https://www.ohrc.on.ca/en/policy-preventing-discrimination-because-gender-identity-and-gender-expression/appendix-b-glossary-understanding-gender-identity-and-expression) Accessed April 18, 2022 2. Torontohealthequity.ca. [Measuring Health Equity: Demographic Data Collection in Health Care](http://torontohealthequity.ca/wp-content/uploads/2018/03/Measuring-Health-Equity-Participant-Manual-2018.pdf). Accessed April 18, 2022 3. Statistics Canada, Government of Canada (2021).[Sex at birth of person](https://www23.statcan.gc.ca/imdb/p3Var.pl?Function=DEC&Id=24101). Accessed April 18, 2022 4. Losty, M. and O’Connor, J. (2017). [Falling outside of the ‘nice little binary box’: a psychoanalytic exploration of the non-binary gender identity](https://www.tandfonline.com/doi/full/10.1080/02668734.2017.1384933). *Psychoanalytic Psychotherapy*, 32(1), pp.40–60 5. Knudson, S. and Hahn, D. (2019). *Committing sociology: critical perspectives on our social world.* Toronto: Pearson Canada. 6. Fewster, P.H. (2018). [Researching for LGBTQ Health](https://lgbtqhealth.ca/community/two-spirit.php). Lgbtqhealth.ca. Accessed April 18, 2022 7. Ucdavis.edu. (2014). [LGBTQIA Resource Center Glossary \| LGBTQIA Resource Center.](https://lgbtqia.ucdavis.edu/educated/glossary) Accessed April 18, 2022 8. Human Rights Campaign (2011). [Glossary of Terms - Human Rights Campaign.](https://www.hrc.org/resources/glossary-of-terms)  Accessed April 18, 2022 |
| 8: Sexual Orientation | **Which category(ies) best describe your sexual orientation? Select all that apply:**   - Asexual or Aromantic - Bisexual - Demisexual - Homosexual or Gay - Heterosexual or Straight - Lesbian - Pansexual - Queer - Two-Spirit (a term by and for Indigenous peoples) - Prefer to self-describe_____________ - Do not know - Prefer not to answer | | Not Applicable | **Why are we asking this question?**  This information lets us know who we serve at the clinic. It helps us understand the range of orientations that people at our clinic may experience.  This question gives you a space to share your orientation and to help avoid assumptions about you or your healthcare.  **Understanding the question:**  **Sexual orientation** is a term for the emotional, physical, romantic, sexual, and spiritual attraction, non-attraction, desire, or affection for another person.^1^  **Aromantic** describes a romantic identity for people who do not experience romantic attraction to people of any gender but may still have sexual attraction to other people.^2^  **Asexual** describes people who do not experience sexual attraction to people of any gender but may still experience romantic attractions to other people.^1,3^  **Bisexual** describes a person whose primary emotional, romantic, or sexual attraction is toward people of both the same and different gender.  **Demisexual** describes a person who feels sexual attraction only to people that they have an emotional bond with.^3^  **Homosexual or Gay** describes a person whose primary emotional, romantic, or sexual attraction is toward people of the same gender.^1^    **Heterosexual or Straight** describes a person whose primary emotional, romantic, or sexual attraction is toward people of an opposite gender.  **Lesbian** describes a person, usually a woman, whose primary emotional, romantic, or sexual attraction is toward people of the same gender.^1^  **Pansexual** describes a person who has emotional, romantic, or sexual desire for people of all genders and sexes.^1^  **Queer** is a term used to express a range of identities and orientations that are beyond the mainstream.^1^  **Two-Spirit** is a term used by Indigenous peoples to self-describe and express diverse sexual identities as having both a masculine and feminine spirit.^4^ It is a way for Indigenous peoples to reconnect with their traditional languages, ways, and cultures.^5^   1. Human Rights Campaign (2011). [Glossary of Terms - Human Rights Campaign.](https://www.hrc.org/resources/glossary-of-terms)  Accessed April 18, 2022 2. Suen, L.W., Lunn, M.R., Katuzny, K., … & Obedin-Maliver, J. (2020). [What Sexual and Gender Minority People Want Researchers to Know About Sexual Orientation and Gender Identity Questions: A Qualitative Study](https://doi.org/10.1007/s10508-020-01810-y). *Archives of Sexual Behavior,* 49(7), pp.2301–2318. 3. Ucdavis.edu. (2014). [LGBTQIA Resource Center Glossary \| LGBTQIA Resource Center.](https://lgbtqia.ucdavis.edu/educated/glossary) Accessed April 18, 2022 4. Knudson, S. and Hahn, D. (2019). *Committing sociology: critical perspectives on our social world.* Toronto: Pearson Canada. 5. Fewster, P.H. (2018). [Researching for LGBTQ Health](https://lgbtqhealth.ca/community/two-spirit.php). Lgbtqhealth.ca. Accessed April 18, 2022 |
| Social Needs |  |  |  |  |
| 9: Education | **What is your current level of education?**   - No formal schooling - Grade school (grade 1-8) - Some high school, but did not graduate - High school or high school equivalency certificate (grade 9-12) - Completed Registered Apprenticeship or other trades certificate or diploma (or ongoing) - College, CEGEP or other non-university certificate or diploma (or ongoing) - Undergraduate degree or some university - Postgraduate degree or professional designation (e.g., Master’s, PhD, MD) - Do not know - Prefer not to answer | | Not Applicable | **Why are we asking this question?**  Your provider may use this information to know how well you are able to read and understand health information such as medication labels and instructions.^1^  Your provider may also use this to connect you with supports that may be helpful to you.  A person’s education level is related to their ability to understand basic health information and access health services. And these may affect their health.^1^  **Understanding the question:**  The term ***‘some’*** in ‘***Some high school***’ means your high school education may be in progress or stopped.  The term ***‘ongoing’*** means schooling or training is in progress.  If you received your education by home-schooling, please select the matching grade level you have completed.   1. Canadian Institute for Health Information (2018) [In Pursuit of Health Equity: Defining Stratifies for Measuring Health Inequality — A Focus on Age, Sex, Gender, Income, Education and Geographic Location.](https://www.cihi.ca/sites/default/files/document/defining-stratifiers-measuring-health-inequalities-2018-en-web.pdf.) Ottawa, ON: CIHI. Accessed April 4, 2022 |
| 10: Income/  Finances | **Do you currently have difficulty paying for basic needs?**   - Yes - No - Not applicable, I do not have to pay for basic needs - Do not know - Prefer not to answer | | Not Applicable | **Why are we asking this question?**  Your provider may use this to refer you to income resources and supports that may be helpful to you.  Low income has been shown to negatively affect mental and physical health.^1-4^    **Understanding the question:**  **Basic Needs** are things that a person requires to achieve and maintain physical and mental well-being and include food, water, shelter, and clothing.^2-4^   1. National Association of Community Health Centre, Inc, Association of Asian Pacific Community Health Organizations, and the Oregon primary Care Association. (2019). [PRAPARE. Protocol for Responding to and Assessing Patients’ Assets, Risks and Experiences. Implementation and Action Toolkit.](https://prapare.org/wp-content/uploads/2021/10/Full-Toolkit.pdf) Accessed April 14, 2022. 2. Collin, C. and Campbell, B. (2008). [Measuring Poverty: A Challenge for Canada](http://www.parl.gc.ca/Content/LOP/ResearchPublications/prb0865-e.htm) Accessed April 14, 2022 3. Sarlo C. (2013[). Poverty: Where do we draw the line?](https://www.fraserinstitute.org/sites/default/files/Poverty-where-do-we-draw-the-line.pdf) Accessed April 13, 2022 4. Homeless Hub. (2021). [Basic Needs](https://www.homelesshub.ca/povertyhub/basic-needs). Accessed April 13, 2022 |
| 11: Food Security | **Please respond to the following statements:**  **a) “Within the past 12 months, we worried whether our food would run out before we could buy or get more”**   - Often True - Sometimes true - Never true - Do not know - Prefer not Answer   b) **“Within the past 12 months, the food we bought just didn’t last and we could not buy or get more.”**   - Often True - Sometimes true - Never true - Do not know - Prefer not Answer | | Not Applicable | **Why are we asking this question?**  Your provider may use this to refer you to resources and supports that may help you obtain food and maintain a healthy diet.^1,2^  Your provider may also use this to give you advice on regular eating and nutrition.^1^  Not having enough food can mean choosing less expensive, more filling, and less healthy food. This can negatively affect your health.^2^  This question was adapted from the Hunger Vital Sign™ screening tool.^3^   1. National Association of Community Health Centre, Inc, Association of Asian Pacific Community Health Organizations, and the Oregon primary Care Association. (2019). [PRAPARE. Protocol for Responding to and Assessing Patients’ Assets, Risks and Experiences. Implementation and Action Toolkit.](https://prapare.org/wp-content/uploads/2021/10/Full-Toolkit.pdf) Accessed April 14, 2022 2. Botelho, FC., Junior, IF., Guerra, LD., Rodrigues, SF., Tonacio, LV. (2020). [Scientific literature on food and nutrition security in primary health care: A scoping review](https://doi-org.uml.idm.oclc.org/10.1080/17441692.2020.1783565). Global Public Health, 15(12), pp. 1902-1916. 3. Hager, E.R., Quigg, A.M., Black, M.M., … & Cook, J.T., de Cuba, S.A.E., Casey, P.H., Chilton, M. and Cutts, D.B., 2010. [Development and validity of a 2-item screen to identify families at risk for food insecurity](https://doi.org/10.1542/peds.2009-3146). *Pediatrics*, *126*(1), pp.e26-e32. |
| 12: Medication Access | **In the past 12 months, were you unable to get medicine or medical supplies, or did you do anything to make them last longer *because of the cost*?**   - Yes - No - Not applicable, I did not have to get any medicine or medical supplies in the past 12 months - Do not know - Prefer not to answer | | Not Applicable | **Why are we asking this question?**  Your provider may use this to refer you to resources that can help you access medicine or medical supplies.  This may also help your provider consider lower cost or free medicine options.  Being able to access and use needed medicine and medical supplies can affect your health.^1^  **Understanding the question:**  **‘Medicine’** includes both prescription and over-the-counter medicines.  **‘Medical supplies’** include any equipment you may need to manage your health or disability. For example, needles, glucose monitors, walking aids.   1. Sobeski, L. M., Schumacher, C. A., Alvarez, N. A., ... & Van Dril, E. (2021). [Medication access: Policy and practice opportunities for pharmacists.](http://doi.org/10.1002/jac5.1373)*Journal of the American College of Clinical Pharmacy*, *4*(1), 113-125. |
| 13: Housing | **a) What is your current housing situation?**   - A place you or your family owns - A place you or your family rents - Social housing, Subsidized housing or Rent-geared-to-income - Supportive housing or Group Home - Long-term care facility - Correctional facility - Staying in someone else’s place because you have no alternative - Experiencing homelessness (e.g., shelter, living in a public place or vehicle) - Other (Specify)_______________ - Do not know - Prefer not to answer | | Not Applicable | **Why are we asking this question?**  Your provider may use this to refer you to resources and supports that may help you with housing needs.  It is known that unstable housing can negatively affect people’s health.^1,2^  **Understanding the question:**  **Social housing** is housing that is subsidized or partly paid for by a level of government.^3^  **Subsidized housing** means receiving help paying for housing from the government or a private organization.^4^  **Rent-geared-to-income** is a type of rent assistance. It lets a person pay a percentage of their income for rent.^5^  **Supportive housing** refers to housing with on-site staff who provide residents with ongoing support with their needs.   1. Krieger, J. and Higgins, D.L. (2002). [Housing and Health: Time Again for Public Health Action. American Journal of Public Health,](https://dx.doi.org/10.2105%2Fajph.92.5.758) 92(5), pp.758–768. 2. Office of Disease Prevention and Health Promotion (2014). [Housing Instability \| Healthy People 2020](https://www.healthypeople.gov/2020/topics-objectives/topic/social-determinants-health/interventions-resources/housing-instability) Healthypeople.gov. Accessed April 7, 2022 3. www.homelesshub.ca. [Affordable Housing \| The Homeless Hub](https://homelesshub.ca/solutions/housing-accommodation-and-supports/affordable-housing). Accessed April 7, 2022 4. Settlement.org. (2018). [What is subsidized housing?](https://settlement.org/ontario/housing/subsidized-housing/subsidized-housing/what-is-subsidized-housing/) Accessed April 7, 2022 5. www.durham.ca. (2021). [Rent-Geared-to-Income Housing (RGI) - Region of Durham](https://www.durham.ca/en/living-here/rent-geared-to-income-housing-rgi.aspx) . Accessed April 7, 2022 |
|  | **b)** **Who do you live with? Select all that apply:**   - Parent(s) or Guardian(s) - Spouse or Partner - Child(ren) - Grandparent(s) - Sibling(s) - Other family - Friends or Roommates - Paid caregiver or attendant - Alone - Other (Specify) ____________________ - Do not know - Prefer not to answer | | Not Applicable | **Why are we asking this question?**  This information may help your provider further understand your living situation and the social supports that may be available to you.  This may also help provide information on aspects of your overall health.  It is known that living with others can impact your health.^1,2^     1. Krieger, J. and Higgins, D.L. (2002). [Housing and Health: Time Again for Public Health Action. American Journal of Public Health,](https://dx.doi.org/10.2105%2Fajph.92.5.758) 92(5), pp.758–768. 2. Office of Disease Prevention and Health Promotion (2014). [Housing Instability \| Healthy People 2020](https://www.healthypeople.gov/2020/topics-objectives/topic/social-determinants-health/interventions-resources/housing-instability) Healthypeople.gov. Accessed April 7, 2022 |
|  | **c) In the past 12 months, was there a time when you were not able to pay the mortgage or rent on time?**   - Yes - No - Not applicable, I do not have to pay mortgage or rent - Do not know - Prefer not to answer | | Not Applicable | **Why are we asking this question?**  Your provider may use this to refer you to housing or financial resources and supports that may be helpful to you.  Not being able to pay your mortgage or rent on time may negatively affect your health (for example, it may cause stress).^1,2^   1. Krieger, J. and Higgins, D.L. (2002). [Housing and Health: Time Again for Public Health Action. American Journal of Public Health,](https://dx.doi.org/10.2105%2Fajph.92.5.758) 92(5), pp.758–768. 2. Office of Disease Prevention and Health Promotion (2014). [Housing Instability \| Healthy People 2020](https://www.healthypeople.gov/2020/topics-objectives/topic/social-determinants-health/interventions-resources/housing-instability) Healthypeople.gov. Accessed April 7, 2022 |
| 14: Transportation | **In the past 12 months, has lack of transportation kept you from medical appointments, meetings, work, or from getting things needed for daily living? Select all that apply:**   - Yes, it has kept me from medical appointments or getting medicines - Yes, it has kept me from non-medical meetings, appointments, work, or getting things that I need - No - Not applicable, I did not need transportation for these activities in the past 12 months - Do not know - Prefer not to answer | | Not Applicable | **Why are we asking this question?**  Your provider may use this to refer you to transportation resources and supports.  Lack of transportation can impact your health.^1-4^ It may make it hard to get to a healthcare appointment on time. It can also make it hard to get to and from work, get healthy food, use medicine on time, or get something you need.  **Understanding the question:**  **“Medical Appointments”** are appointments to meet your health needs. This may include an appointment with your doctor(s), specialist, or social worker. This also includes getting medicine from your doctor or pharmacist.^1^  You might lack transportation because of:^4^   - cost and access, - distance, - gas or parking costs, - public transportation safety, - and/or other reasons.   **“Things needed for daily living”** are things such as groceries or personal products that you need to be healthy.  This question was adapted from the PRAPARE tool.^1^   1. National Association of Community Health Centre, Inc, Association of Asian Pacific Community Health Organizations, and the Oregon primary Care Association. (2019). [PRAPARE. Protocol for Responding to and Assessing Patients’ Assets, Risks and Experiences. Implementation and Action Toolkit.](https://prapare.org/wp-content/uploads/2021/10/Full-Toolkit.pdf) Accessed April 14, 2022 2. Billioux, A., Verlander, K., Anthony, S., Alley, D. [Standardized screening for health-related social needs in clinical settings: the accountable health communities screening tool](https://doi.org/10.31478/201705b). Discussion Paper, National Academy of Medicine, Washington, DC. Accessed April 14, 2022 3. Razon, N., Gottlieb, L. (2022). [Content Analysis of Transportation Screening Questions in Social Risk Assessment Tools: Are we capturing transportation insecurity](https://doi.org/10.3122/jabfm.2022.02.210256). Journal of the American Board of Family Medicine, 35(2), pp.400-405. 4. Syed, ST., Gerber, BS., Shapr, LK. (2013[). Traveling toward disease” transportation barriers to health care access](https://doi.org/10.1007/s10900-013-9681-1). J community Health, 38(5), pp. 976-993. |
| 15: Phone and Internet Access | **Do you currently have consistent access to a phone or the internet?**   - Yes, phone only - Yes, internet only - Yes, both - No - Do not know - Prefer not to answer | | Not Applicable | **Why are we asking this question?**  Your provider may use this to refer you to resources that can improve your access to phone and internet services.  These resources may help improve your access to health care appointments and health information.  This information may also inform how you receive health care services. For example, by phone, video, or in-person.  Access to a phone and internet is now a basic human right. This is due to its impact on access to health care and other needed services.^1,2^  **Understanding the question:**  **“Consistent access”** means having regular, reliable, and adequate access to a phone or internet to obtain needed services. This includes access to a phone or internet for a healthcare appointment.^3^ This also includes access to health information that is on the internet that your provider refers you to.^3^   1. Benda, NC., Veinot, TC., Sieck, C., Ancker, JS. (2020). [Broadband internet access is a social determinant of Health!](https://doi.org/10.2105/AJPH.2020.305784) AM J Public Health, 110(8), pp. 1123-1125. doi: 2. Rubin, R., 2021[. Internet access as a social determinant of health.](https://doi.org/10.1001/jama.2021.11733) *JAMA*, *326*(4), pp.298-298. 3. Graves, JM., Abshire, DA., Amiri, S., Mackelprang, JL. (2021). [Disparities in technology and broadband internet access across rurality: implications for health and education.](https://doi.org/10.1097/fch.0000000000000306) Fam Community Health, 44(4), pp. 257-265. |
| 16: Utilities | **In the past 12 months, did you miss making a payment on any utility bills (e.g., electric, gas/oil, water) *because of cost*?**   - Yes - No - Not applicable, I did not have to pay utility bills in the past 12 months or utilities already included in rent - Do not know - Prefer not to answer | | Not Applicable | **Why are we asking this question?**  Your provider may use this to refer you to supports that may be helpful to you.  Difficulty paying for utilities can negatively affect your health.^1^ For example, you need heat when it is cold outside.^2^  Difficulty paying utility bills can also be a sign of financial need, which can affect your health (for example, it may cause stress).  **Understanding the question:**  This question is asking about basic utilities. Basic utilities may include:   - heating or cooling - electricity or hydro - water or sewer - phone, and internet.   The specific utilities you pay for may depend on where you live. For example, you may heat your home using natural gas, furnace oil or wood depending on where you live.   1. International Policy Centre for Inclusive Growth (2009) [Equitable access to basic utilities: Public versus private provision and beyond](https://www.undp.org/library/equitable-access-basic-utilities-public-versus-private-provision-and-beyond). Accessed: April 20, 2022. 2. Grey, C. N., Jiang, S., Nascimento, C., Rodgers, S. E., Johnson, R., Lyons, R. A., & Poortinga, W. (2017). [The short-term health and psychosocial impacts of domestic energy efficiency investments in low-income areas: a controlled before and after study](https://doi.org/10.1186/s12889-017-4075-4). BMC Public Health, 17(1), 1-10. |
| 17: Social Supports | **a) Do you feel you have people who you can open up to or confide in?**   - Yes, I always or sometimes have someone - No, I don’t have anyone - Do not know - Prefer not to answer   **b) Do you have people to rely on if you needed help?**   - Yes, I always or have someone - No, I don’t have anyone - Do not know - Prefer not to answer | | Not Applicable | **Why are we asking this question?**  Your provider may use this to refer you to social resources and social supports that may be helpful to you.  **Understanding the question**  Having “**people you feel you can open up to or confide in*”*** means being able to talk to people that you care about, trust and feel close to.^1^  Having social relationships and feeling connected can impact health.^1^  **b. Why are we asking this question?**  Your provider may refer you to social resources and social supports that may be helpful to you.  Having **“people you can rely on*”*** can help you to feel more socially connected and secure, and this may impact your health.   1. National Association of Community Health Centre, Inc, Association of Asian Pacific Community Health Organizations, and the Oregon primary Care Association. (2019). [PRAPARE. Protocol for Responding to and Assessing Patients’ Assets, Risks and Experiences. Implementation and Action Toolkit.](https://prapare.org/wp-content/uploads/2021/10/Full-Toolkit.pdf) Accessed April 14, 2022 |
| 18: Employment | **a) Are you currently employed (this includes self-employed, full-time, part-time or other)?**   - Yes - No - Do not know - Prefer not to answer | | **a) Are you currently employed (this includes self-employed, full-time, part-time or other)?**   - Yes → Go to question 18c - No → Go to question 18b - Do not know → Go to question 18b - Prefer not to answer → Go to question 18b | **Why are we asking this question?**  Your provider may use this information to better understand employment circumstances that could be affecting your health. Your provider may also refer you to employment resources that could be helpful. It is known that those who work tend to have better health than those who are unemployed.^1^   1. Waddell, G., & Burton, A. K. (2006). [Is work good for your health and well-being?](https://cardinal-management.co.uk/wp-content/uploads/2016/04/Burton-Waddell-is-work-good-for-you.pdf) Accessed April 4, 2022. |
|  | **b)** **Are you currently looking for work?**   - Yes - No - Not applicable, I am currently employed [remove if skip logic is used] - Do not know - Prefer not to answer | | b) [If no is selected in 18a] **Are you currently looking for work?**   - Yes → Go to question 19 - No → Go to question 19 - Do not know → Go to question 19 - Prefer not to answer → Go to question 19 | **Why are we asking this question?**  Your provider may use this information to refer you to employment resources that may be helpful. |
|  | c) **Is your main job temporary or part-time (e.g., casual, contract, freelance, short-term, seasonal)?**   - Yes - No - Not applicable, I am not currently employed [remove if skip logic is used] - Do not know - Prefer not to answer | | c) [If yes is selected in 18a] **Is your main job temporary or part-time (e.g., casual, contract, freelance, short-term, seasonal)?**   - Yes → Go to question 18d - No → Go to question 18d - Do not know → Go to question 18d - Prefer not to answer → Go to question 18d | **Why are we asking this question?**  Your provider may use this information to better understand employment circumstances that may be affecting your health.  Your provider may also refer you to employment resources that could be helpful.  It is known that unstable or unpredictable work negatively affects peoples’ health.^1,2^  **Understanding the question:**  **“Main job”**: If you have more than one job, your main job is your primary source of income.   1. Benach, J., Vives, A., Amable, M., Vanroelen, C., Tarafa, G. and Muntaner, C., 2014. [Precarious employment: understanding an emerging social determinant of health.](https://doi.org/10.1146/annurev-publhealth-032013-182500)*Annual review of public health*, *35*, pp.229-253. 2. Benach, J., Vives, A., Tarafa, G., Delclos, C. and Muntaner, C., 2016. [What should we know about precarious employment and health in 2025? Framing the agenda for the next decade of research.](https://doi.org/10.1093/ije/dyv342)*International journal of epidemiology*, *45*(1), pp.232-238. |
|  | **d)** **Do you feel that your current employment could be negatively affected if you raised concerns about your work (e.g., health, safety, rights)?**   - Yes - No - Not applicable, I am not currently employed [remove if skip logic is used] - Do not know - Prefer not to answer | | [If yes is selected in 18a] **Do you feel that your current employment could be negatively affected if you raised concerns about your work (e.g., health, safety, rights)?**   - Yes → Go to question 18e - No → Go to question 18e - Do not know → Go to question 18e - Prefer not to answer → Go to question 18e | **Why are we asking this question?**  Your provider may use this information to better understand employment circumstances that may be affecting your health.  Your provider may also refer you to employment resources that could be helpful.  A person’s inability or unwillingness to raise concerns about health, safety and rights at work can negatively affect their health.^1^  **Understanding the question:**  **Health and safety concerns** at work may include concerns about unsafe work (for example, lifting items that are too heavy, working with violent clients), unsafe work environments (for example, slippery floors, broken machinery), or harassment due to race, gender, or sexual orientation.^1^  **Concerns about employment rights** may include questions about your right to breaks, sick leave or vacation time.^1^  Your **health and safety rights^1^** include your right to**:**   - know about unsafe work - refuse unsafe work - participate in workplace health and safety decisions  1. Lewchuk, W., 2013. [The limits of voice: Are workers afraid to express their health and safety rights.](https://digitalcommons.osgoode.yorku.ca/cgi/viewcontent.cgi?article=1001&context=ohlj) Osgoode Hall LJ, 50(4), p. 789-812. Accessed April 20, 2022 |
|  | e) **In the past 12 months, did your income change a lot from month to month?**   - Yes - No - Not applicable, I am not currently employed [remove if skip logic is used] - Do not know - Prefer not to answer | | [If yes is selected in 18a] **In the past 12 months, did your income change a lot from month to month?**   - Yes → Go to question 19 - No → Go to question 19 - Do not know → Go to question 19 - Prefer not to answer → Go to question 19 | **Why are we asking this question?**  Your provider may use this information to understand employment conditions that could be affecting your health.    Your provider may also refer you to employment or income resources that could be helpful.  Changing income is a key feature of unstable or unpredictable work. This is known to have negative effects on health^1^.   1. Lewchuk, W., 2012. [The limits of voice: Are workers afraid to express their health and safety rights.](https://digitalcommons.osgoode.yorku.ca/cgi/viewcontent.cgi?article=1001&context=ohlj) Osgoode Hall LJ, 50, p. 789-812. Accessed April 20, 2022 |
| Optional Questions |  |  |  |  |
| 1: Ethnicity | **What is your ethnic or cultural background?** e.g., Chinese, Filipino, Guyanese, Scottish, Somali, Korean __________________ | | Not Applicable | **Why are we asking this question?**  This question gives people a space to share their ethnic or cultural identity. This information lets us know who we serve at the clinic and may help us understand and address the unique needs of our patients.  **Understanding the question:**  This question on ethnicity should not be confused with Race. **Ethnicity** refers to groups of people who share a common culture or ancestry. They may also share a common language, religion, geographic origin, nationality, cultural traditions, migration history and so on.^1,2^  **Race** is a term used to classify people into groups usually based on observable physical characteristics (e.g., skin colour) but can also include characteristics such as accent, dress and so on.^1,2,3^  Although these differences or groupings are created  by the society we live in, racial categories can have significant consequences for people’s lives, including unfair and unjust treatment by individuals and institutions. This is what we call racism.  We welcome you to share the ethnic group or culture you identify with.   1. Canadian Institute for Health Information (2022) [Guidance on the Use of Standards for Race-Based and Indigenous Identity Data Collection and Health Reporting in Canada](https://www.cihi.ca/sites/default/files/document/guidance-and-standards-for-race-based-and-indigenous-identity-data-en.pdf) Ottawa, ON: CIHI. Accessed April 5, 2022 2. Torontohealthequity.ca. [Measuring Health Equity: Demographic Data Collection in Health Care](http://torontohealthequity.ca/wp-content/uploads/2018/03/Measuring-Health-Equity-Participant-Manual-2018.pdf). Accessed April 5, 2022 3. Government of Ontario Anti-Racism Directorate. [Data Standards for the Identification and Monitoring of Systemic Racism](https://www.ontario.ca/document/data-standards-identification-and-monitoring-systemic-racism). Accessed April 5, 2022 |
| 2: Religion | **What is your religious or spiritual affiliation? Select all that apply:**   - Agnosticism - Animism or Shamanism - Atheism - Baha’i Faith - Buddhism - Christian Orthodox - Christian, *not included elsewhere on this list* - Confucianism - Hinduism - Islam - Jainism - Judaism - Native Spirituality - Pagan - Protestant - Rastafarianism - Roman Catholic - Sikhism - Spiritual - Unitarianism - Zoroastrianism - Other (Specify) _____________________ - Not Applicable, I do not have a religious or spiritual affiliation - Do not know - Prefer not to answer | | Not Applicable | **Why are we asking this question?**  Your provider may use this to better understand how your religious or spiritual beliefs and practices influence your health or health behaviors. This may include your diet, holidays you observe, or the health care treatment options you feel comfortable with.  Your provider may use this to inform the care you receive.  It is known that religious affiliation or involvement can positively affect health^1^.  **Understanding the question:**  “**Religious or spiritual affiliation**” is about your connection with a particular set of beliefs, attitudes, or practices. This may or may not be related to a specific religion (for example, Catholicism, Islam) or belief in a higher being.^1^ This also relates to a person's sense of spirituality.  Select the religion(s) you feel connected to. This includes a religion you are actively practicing, your family raised you in, or you feel close to.  You can select more than one option. If you do not find your religion listed, you can write it in.   1. Chatters, L. (2000) [‘Religion and Health: Public health research and practice,’](https://doi.org/10.1146/annurev.publhealth.21.1.335) Annual Review of Public Health, 21, pp. 335-367. Doi: |
